# A DNA Methylation Signature of Addiction in T Cells and Its Reversal With DHEA Intervention

**DOI:** 10.3389/fnmol.2018.00322

**Published:** 2018-09-10

**Authors:** Elad Lax, Gal Warhaftig, David Ohana, Rachel Maayan, Yael Delayahu, Paola Roska, Alexander M. Ponizovsky, Abraham Weizman, Gal Yadid, Moshe Szyf

**Affiliations:** ^1^Department of Pharmacology and Therapeutics, McGill University, Montreal, QC, Canada; ^2^The Mina and Everard Goodman Faculty of Life Sciences, Bar-Ilan University, Ramat Gan, Israel; ^3^Max Wertheimer Minerva Center for Cognitive Processes and Human Performance, Technion – Israel Institute of Technology, Haifa, Israel; ^4^Laboratory of Biological Psychiatry, Felsenstein Medical Research Center, Research Unit and Geha Mental Health Center, Tel Aviv University, Tel Aviv, Israel; ^5^Yehuda Abarbanel Mental Health Center, Bat Yam, Israel; ^6^Department for the Treatment of Substance Abuse and Mental Health Services, Israeli Ministry of Health, Jerusalem, Israel; ^7^The Hebrew University of Jerusalem, Jerusalem, Israel; ^8^The Leslie and Susan Gonda (Goldschmidt) Multidisciplinary Brain Research Center, Bar-Ilan University, Ramat Gan, Israel; ^9^Program for Epigenetics and Psychobiology, McGill University, Montreal, QC, Canada

**Keywords:** dehydroepiandrosterone (DHEA), DNA methylation, drug abuse, genome-wide analysis, drug-addiction

## Abstract

Previous studies in animal models of cocaine craving have delineated broad changes in DNA methylation profiles in the nucleus accumbens. A crucial factor for progress in behavioral and mental health epigenetics is the discovery of epigenetic markers in peripheral tissues. Several studies in primates and humans have associated differences in behavioral phenotypes with changes in DNA methylation in T cells and brain. Herein, we present a pilot study (*n* = 27) showing that the T cell DNA methylation profile differentiates persons with a substance use disorder from controls. Intervention with dehydroepiandrosterone (DHEA), previously shown to have a long-term therapeutic effect on human addicts herein resulted in reversal of DNA methylation changes in genes related to pathways associated with the addictive state.

## Introduction

Several lines of evidence suggest a crucial role for epigenetic modification in forming and maintaining the drug-addicted state (Maze and Nestler, 2011; Xu et al., 2016). One of the basic epigenetic mechanisms is DNA methylation, the covalent binding of a methyl group to cytosine to form 5-methylcytosine. The DNA methylation landscape was shown to be altered in different animal models for drug addiction (Maze and Nestler, 2011; Itzhak et al., 2015; Wright et al., 2015). Human studies have focused mainly on the use and craving of alcohol, in which differentially methylated sites were associated with alcohol abuse in a genome-wide analysis of whole-blood samples (Ruggeri et al., 2015), and low methylation levels of the leptin promoter was associated with higher alcohol craving (Hillemacher et al., 2015). However, the state of peripheral DNA methylation in abusers of substances other than alcohol is yet unclear.

Dehydroepiandrosterone (DHEA), an endogenous neurosteroid, negatively modulates the levels of the stress hormone cortisol (Flood et al., 1988), reduces anxiety and restores stable mood, as demonstrated both in animal models (Melchior and Ritzmann, 1994; Genud et al., 2009) and in humans (Morales et al., 1994; Wolkowitz et al., 1999). Administration of DHEA to rodents reduces cocaine intake and cocaine-seeking behavior during extinction and cocaine-primed reinstatement (Doron et al., 2006; Maayan et al., 2006). In humans, reduced DHEA levels during drug abstinence were found to predict later drug reuse (Buydens-Branchey et al., 2002; Wilkins et al., 2005). Moreover, a recent double-blind, placebo-controlled study found that DHEA add-on during an in-patient drug rehabilitation program reduced both negative affects during treatment and drug relapse in a 16-month follow-up (Ohana et al., 2016).

Previous reports showed that DHEA administration alters DNA methylation in animal models (Prasanna et al., 1989; Zhu et al., 2010). Also, DHEA is known to reduce stress (Flood et al., 1988) and DNA methylation is extensively studied as an epigenetic underlying mechanism of stress response (Booij et al., 2013). Therefore, we hypothesized that the beneficial effects of DHEA on addiction are mediated, at least partially, through alteration of DNA methylation.

As DNA methylation cannot be assessed in the brain of living humans, peripheral DNA methylation patterns are extensively being used in recent years as an indication for the epigenetic footprint of psychiatric disorders. The immune system is of particular interest due to its crosstalk with the brain. Indeed, whole blood and T-cell DNA methylation patterns have been shown to correlate with several psychiatric disorders; we and others have demonstrated that conditions such as early-life stress, depression and post-traumatic stress disorder (PTSD) alter DNA methylation across tissues in T-cells and brain tissue and across species (Provencal et al., 2012; Labonte et al., 2014; Nieratschker et al., 2014; Nemoda et al., 2015; Oh et al., 2015).

In the current study, we focused on DNA methylation in human CD3^+^ T-cells, as a central cell in the peripheral immune system, and to reduce confounding effects presented by cellular heterogeneity of whole blood samples (Jaffe and Irizarry, 2014). Blood samples were obtained before and 1 month after treatment initiation from poly-drug users in an inpatient rehabilitation program, who were included in a double-blind placebo-controlled DHEA add-on study. Both the placebo and DHEA-treated group were drug clean throughout the 1-month treatment period, and samples were submitted for methylation profiling only from individuals who did not reuse drugs (for the DHEA group) up to the 16-month follow-up. Healthy controls, recruited from the general population in the same geographical region of the rehabilitation center, did not use any drug during their lifetime. We delineated the genome-wide DNA methylation signature of drug addiction in peripheral T cells by comparing DNA methylation profiles of addicts and healthy controls. This study may enable finding common early epigenetic markers that could be used to predict treatment effectiveness across a wide range of poly-drug use patterns. In addition, we show that DHEA treatment which reduces drug-abuse relapse also reverses the altered DNA methylation pattern of several genes in pathways related to neurotransmission and corticotrophin release.

## Materials and Methods

### Participants and Clinical Data

Nineteen poly-drug users from the Malkishua Drug Rehabilitation Center, Israel, participated in the study. These subjects had participated in the clinical study described in detail in Ohana et al. (2016). The study was approved by the Helsinki Committee of Abarbanel Mental Health Center (Bat Yam, Israel) and by the Israeli Ministry of Health (proposal no. #341). Inclusion criteria were the diagnosis of substance abuse and the provision of a written informed consent. Exclusion criteria included age below 18 and over 50; serious kidney, lung, liver, neurological, prostatic or cardiovascular diseases; and suicide risk, acute psychosis, psychotic disorder, bipolar disorder, severe depressive episode or organic brain syndrome, as well as HIV or hepatitis C. Participants underwent physical examination by a licensed physician to assess their general health condition. All participants were diagnosed by a senior psychiatrist using DSM-IV-TR criteria, and all were found to have a substance use disorder. For all but one participant, the positive diagnosis was for at least one drug other than alcohol. Nine healthy controls were recruited from the general population living in the same geographical region of the rehabilitation center, who self-reported to have never used any drug of abuse.

The age and sex distribution is presented in **Table 1**. There was no statistical difference in age or sex between the groups. The drugs of abuse that were consumed by the participants are summarized in **Table 2** (see **Supplementary Table 1** for drug intake by each individual). Each patient underwent stabilization from drug effects (i.e., detoxification) during the first 7 days after arrival at the rehabilitation center, and signed an informed consent form to participate in the study, provided by the treating doctor. The placebo-controlled, double-blind, randomized clinical trial with the DHEA add-on treatment (given in addition to the standard program at the center which included intensive psychosocial interventions and aftercare) began 1 week after arrival at the center. Participants received placebo or DHEA supplement for 1 month and abstained from drug use during this period. Blood was drawn from the participants before treatment and 1 month after treatment. The center’s social worker evaluated each participant for relapse approximately 16 months (16.3 ± 1.0) following treatment at the rehabilitation center. An evaluation of ‘no relapse’ was based on two obligatory criteria: (1) the participant’s self-report of no drug use whatsoever since the release from the rehabilitation center and up to the time of the interview; (2) for patients who resided in hostels after release, provision of a report from the hostel staff, affirming complete abstinence from drug use during the 16-month period. Samples from participants were processed and analyzed only after confirming that DHEA-treated subjects did not use drugs from the initiation of treatment (*n* = 10) up to 16 months after treatment, while placebo-treated subjects reported drug re-use (*n* = 9). Samples from DHEA treated patients who relapsed to drug use were not analyzed in this study.

**Table 1:** Demographic characteristics of the different groups.

	*n*	Age	Gender
DHEA	10	25.6 ± 2.57	8M/2F
Placebo	9	22 ± 1.31	7M/2F
Healthy controls	9	18.5 ± 0.3	9M
*p*-value		*p* > 0.05 (not significant)	

**Table 2:** Drugs of abuse consumed by the participants.

Drug	DHEA	Placebo
Cannabis	10	8
Synthetic cannabinoids	1	2
Alcohol	1	3
LSD	2	3
Opiates (Heroin/Methadone/Opium)	2	3
Cocaine	1	0
Amphetamine/Methamphetamine/Cathinone	0	2
Benzodiazepines	2	0
MDMA	1	5
Methylphenidate	1	0

### DHEA Administration Within the Treatment Program

DHEA and placebo were purchased from Bio Synergy Health Alternatives (Boise, ID, United States). DHEA and placebo treatments were entrusted to a staff member (nurse) of the rehabilitation center and orally administered in a double-blind manner to each patient. Each capsule had one of four possible colors (two containing placebo and two containing DHEA). As per the double-blind procedure, neither the experimenter nor the nurses were aware of the association between experimental condition and capsule color. One DHEA capsule (100 mg) was administered once a day in the morning after breakfast. The dose of 100 mg DHEA/day is the minimum therapeutic amount recommended by the manufacturer in order to avoid adverse effects (Strous et al., 2005). All adverse effects reported by the study participants were assessed for severity and relationship to the medication. At baseline, all patients were instructed to abstain from use of drugs that would potentially suppress their craving for substances of abuse (including benzodiazepines, antidepressants, metadoxine, naltrexone, acamprosate) during the study and follow-up period. On blood collection days, all participants were further instructed to avoid morning exercises, caffeine consumption and smoking, which could affect morning cortisol or DHEA/S levels, until blood sampling was completed. All participants received all other treatments and psychosocial care regularly provided by the drug-rehabilitation center.

### Psychological Tests

To assess mood and well-being, participants completed the Positive and Negative Affect Scale (PANAS; Watson et al., 1988). Additionally, the effect of treatment on decision making was examined using the Iowa Gambling Task (IGT; Bechara et al., 1994). In this decision task, participants repeatedly select among four decks of cards that yield monetary outcomes. Two of the decks are advantageous, producing small gains and smaller losses, and leading to net (accumulating) gains. The other two decks are disadvantageous, yielding larger gains but much steeper losses, which result in net losses. The payoff structure of the task is described in **Supplementary Table 2**.

### Illumina Beadchip 450K Analysis

DNA was extracted from T cells isolated using anti-CD3 immuno-magnetic beads (Dynabed Life Technologies), bisulfite converted and subjected to Illumina HumanMethyaltion450K BeadChip analysis. Samples were randomized with respect to slide and position on arrays and all samples were hybridized and scanned concurrently to mitigate batch effects as recommended by McGill genome center using Illumina Infinum HD technology user guide. Illumina arrays were analyzed using the ChAMP Bioconductor package in R (Morris et al., 2014). IDAT files were used as input in the champ.load function using minfi quality control and normalization options. Raw data were filtered for probes with a detection value of *P* > 0.01 in at least one sample. We filtered out probes on the X or Y chromosome to mitigate sex effects and probes with SNPs as identified in Marzouka et al. (2016), as well as probes that align to multiple locations as identified in Marzouka et al. (2016). Batch effects were analyzed on the non-normalized data using the function champ.svd. Intra-array normalization to adjust the data for bias introduced by the Infinium type 2 probe design was performed using beta-mixture quantile normalization (BMIQ) with function champ.norm (norm = “BMIQ”) (Morris et al., 2014). We corrected for batch effects after BMIQ normalization using champ.runcombat function. Differentially methylated CGs (MVP) were called using the Bioconductor package Limma (Smyth et al., 2005) as implemented in ChAMP using “fdr” for multiple testing correction [adjusted *P*-value (Q) of <0.05]. ANOVA analysis was performed for CGs that were short listed for association with addiction using “loop_anova lmFit” function with Bonferroni adjustment for multiple testing. Following analysis, large differences in DNA methylation were observed between the DHEA pre-treatment group (*n* = 9) and all other groups which could not be explained by any of the confounding variables. These large differences most probably represent a technical error in the Illumina Beadchip assay and we therefore removed these samples from our analysis and performed genome-wide analysis only for samples from healthy controls and placebo-treated addicts before treatment. Hierarchical clustering was performed using one minus Pearson correlation and heatmaps were generated using the Broad institute GENE-E application. Next, we analyzed differentially methylated probes which were annotated to promoters of known genes with Ingenuity Pathway Analysis (IPA) software (Qiagen). In total, out of 5,532 differentially methylated CpGs with FDR-corrected *p*-value < 0.05, 126 hyper-methylated and 829 hypo-methylated CpGs annotated to gene promoters were analyzed. The aim of the IPA analysis was to screen and discover pathways which are known to be relevant to mental health, neuronal and brain function or addiction. Because IPA analysis was performed on FDR-corrected significantly differentially methylated CpGs and was used as a screening tool for pathways of interest, we avoided an additional FDR correction for pathway analysis which can result in overcorrection and high rates of false-negative results.

Based on the IPA analysis, we found two interesting pathways with hyper-methylated CpGs: “Reelin signaling in neurons” (*p* = 0.00741) and “Corticotrophin releaseing hormone Signaling” (*p* = 0.02291). We also found four relevant pathways with hypo-methylated CpGs: “Noradrenaline and adrenaline degradation” (*p* = 0.00195), “Ethanol degradation” (*p* = 0.00724), “serotonin degradation” (*p* = 0.00891) and “dopamine degradation” (*p* = 0.01585). Since genes in these pathways are highly overlapping, we hereafter call this group of genes in the more general term “cathecholamines, serotonin, and ethanol degradation.”

Next, we explored the effect of 1 month DHEA or placebo treatment on DNA methylation of these CpGs, compared to healthy controls using one-way ANOVA followed by Tukey *post hoc* analysis and reported the nominal *p*-values of the significant *post hoc* tests.

In addition, we analyzed the correlation between DNA methylation levels of patients and their PANAS score and IGT score. Healthy individual did not fill questionnaires, therefore we analyzed data from placebo-treated at baseline and after 1 month of placebo treatment as well as data from DHEA-treated patients (only after treatment, as explained above). The rationale of this analysis was to consider the relation between DNA methylation and well being score and decision-making behaviors regardless of treatment group. We performed individual Pearson’s correlation coefficient analysis for each CpG in the Illumina array and FDR-corrected for multiple testing. Although individual correlations of DNA methylation and PANAS and IGT score for each group is of interest, it could not be properly performed in this study because the limited sample size of each group will not allow any *p*-value to stand multiple-test correction. This is based on a simulation we did with a samples size of 9 and Pearson’s *r*-values of up to 0.99 followed by FDR correction for 450000 CpGs.

### Comparative Analysis to Nucleus Accumbens DNA Methylation Patterns in Rat Model of Drug Addiction

Currently, there are not widely used and acceptable animal models for poly-drug abuse.

Therefore, we focused our across-species and across-tissues analysis on genome-wide DNA methylation data previously obtained and validated from experiments with rats trained to self-administer intra-venous cocaine. Our hypothesis is that there is a common signature for the “drug abusive phenotype” irrespective of the drug abused that will be revealed by such a comparison. We also hypothesize the possibility of DNA methylation differences that are unique for cocaine consumption and cocaine abuse that will not overlap with the poly-drug abusers, most of which were not exposed to cocaine. Surgeries, behavioral experiments and tissue processing are detailed in our previous study (Massart et al., 2015). In brief, male Sprague-Dawley rats (Harlan) weighing 250–350 g were maintained on a 12 h light/12 h dark reversed cycle, with food and water available *ad libitum*. All experimental procedures were approved by the University Animal Care and Use Committees and were performed in accordance with National Institutes of Health guidelines. Rats were anesthetized and implanted with intravenous silastic catheters into the right jugular vein. The catheter was secured to the vein, was passed subcutaneously to the top of the skull, where it exited into a connector, mounted to the skull with screws and dental cement. Carprofen (2 mg/kg; SC) was administered post-surgery to relieve pain. Rats were trained to self-administer cocaine for 6 h per day, over 10 d (0.75 mg/kg, 0.13 ml, 5 s/infusion; cocaine obtained from the National Institutes on Drug Abuse). Rats had 1 day of withdrawal, and then were sacrificed (*n* = 8/group), their brains removed, and tissue punches were taken for NAc isolation and Methylated DNA immune-precipitation (MeDIP)-array analysis as described before (Massart et al., 2015).

Comparison of differentially methylated gene lists between human and rat experiments was performed using Venny web tool^1^. Significance of overlap between two groups was determined using hypergeometric Fisher exact test.

## Results

### Broad Signature of DNA Methylation Distinguishes Addicts From Healthy Controls

**FIGURE 1:**
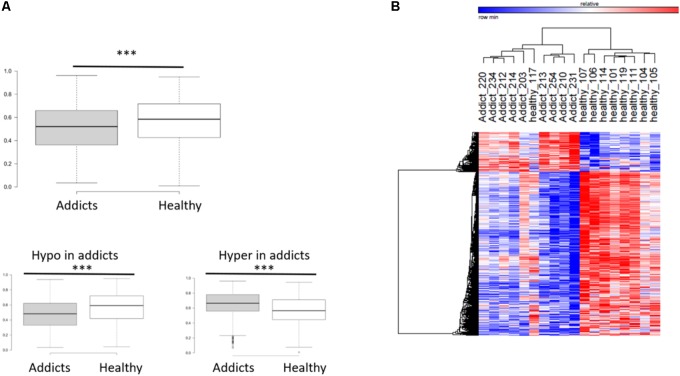
DNA methylation differences associated with addiction. **(A)** Box plot of average methylation of sites that are differentially methylated between addicted and healthy controls. Bottom panel: the average methylation of genes that become less methylated (hypomethylation) and more methylated (hypermethylation) in addicts ^∗∗∗^*p* < 2.2E-16; Mann–Whitney test. **(B)** Heat map showing clustering by one minus Pearson correlations of healthy controls and addicts using the 5,532 differentially methylated genes (4,458 probes were hypomethylated and 1,074 hypermethylated in addicts).

To compare between the DNA methylation profiles of patients (placebo group before treatment) and healthy controls, we performed a genome-wide measurement of DNA methylation in ∼480K CpGs using the Illumina Infinium Human Methylation 450K BeadChip Array platform, as described in “Materials and Methods” section. After quality control, 423,956 DNA methylation loci were analyzed, and differentially methylated CGs between healthy controls and addicts (placebo group before treatment) were delineated using the Bioconductor package Limma (Smyth et al., 2005) as implemented in ChAMP. We found 5,532 CGs differentially methylated between healthy controls and the patients (FDR < 0.05). Out of which 4,458 CGs were hypo-methylated and 1,074 were hyper-methylated. A box plot of the differentially methylated sites reveals a highly significant overall hypomethylation of the differentially methylated sites in addicts as compared to healthy controls (**Figure 1A**). Hierarchical clustering by one minus Pearson correlation for the differentially methylated sites reveals extensive hypomethylation in the addicted group. These sites cluster healthy controls and addicts in separate groups, with the exception of one healthy person (heatmap, **Figure 1B**). These data suggest a broad signature of addiction in DNA methylation of T cells.

Since almost all the patients were smokers, we inspected the data for differentially methylated CpGs which may be caused by nicotine smoking. We created a list of 128 CpG sites (**Supplementary Table 3**) which were found to be significantly differentially methylated in at least one of five Illumina 450K BeadChip array DNA studies (Zeilinger et al., 2013; Dogan et al., 2014; Tsaprouni et al., 2014; Teschendorff et al., 2015; Zhu et al., 2016). Out of 5,532 CpGs found in the present study, 14 CpGs were overlapped with smoking (*p* = 1.76E-9, hyper-geometric test). This finding suggest that our study was capable to replicate some of the effects of smoking on DNA methylation. However, a large majority of our differentially methylated CpGs are not unique to smoking, this is most probably because previous studies had older participants (most of them at an age range of 40–60) with a long history of smoking, while our cohort is of young addicts. Therefore, it is very probable that some of the CpGs associated with smoking are differentially methylated also as a function of age and possibly also represent the long-term toxic effect of smoking rather than the addictive properties of nicotine *per se*. We concluded that changes in DNA methylation in our study are mostly due to drug addiction including the addictive effects of nicotine.

### Functional Footprint of Differentially Methylated Genes

**FIGURE 2:**
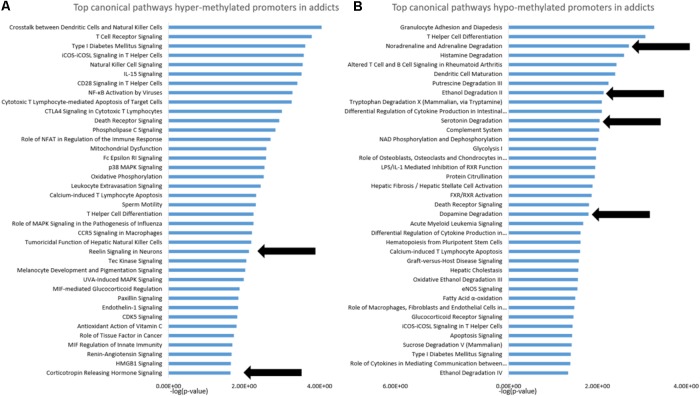
Functional footprint of differentially methylated gene promoters in addicts. **(A)** Canonical gene-pathways that are enriched with genes that are hypermethylated in addicts. **(B)** Canonical gene-pathways that are enriched with genes that are hypomethylated in addicted persons. Arrows indicate neurobiological-related pathways further analyzed for the effect of DHEA.

To gain insight into the functional footprint of the differentially methylated genes in T cells from addicts and healthy subjects, the short list of gene-annotated promoters generated from the differential methylation analyses were subjected to a gene set enrichment analysis using IPA software (**Figures 2A,B**). Interestingly, many of the pathways enriched in genes whose promoters were differentially methylated in addicts are involved in immunity and inflammation, which is consistent with a change in T cell immune function and inflammation in the addicted group. Notably, however, several neurobiologically related pathways were enriched in both genes that are hypermethylated (reelin and corticotropin release) and hypomethylated (noradrenaline degradation, ethanol degradation, serotonin degradation, and dopamine degradation) in addicted individuals.

### Reversal of Differentially Methylated Neurobiologically Related Genes by DHEA Supplement Intervention

**FIGURE 3:**
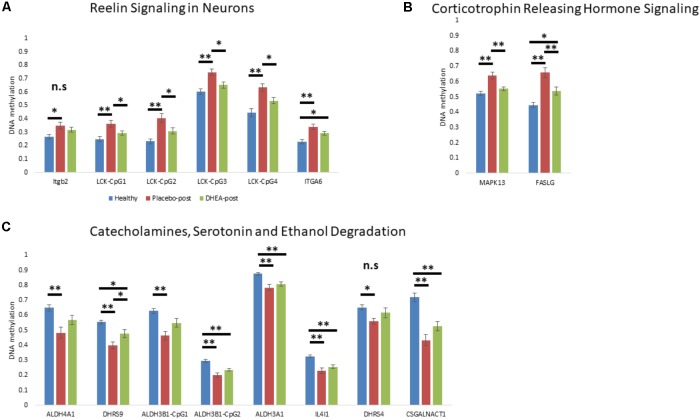
The effect of DHEA intervention on neurobiological-related gene promoters. **(A)** Gene promoters hypermethylated in addicts and related to the “Reelin Signaling in Neurons” pathway. **(B)** Gene promoters hypermethylated in addicts and related to the “Corticotrophin Releasing Hormone Signaling” pathway. **(C)** Gene promoters hypomethylated in addicts and related to the “Catecholamines, Serotonin, and Ethanol Degradation” pathway. Methylation values were determined using Illumina 450K arrays. Statistical significance was determined using one-way ANOVA analyses followed by Bonferroni correction for multiple testing and Tukey *post hoc* test. ^∗^*p* < 0.05, ^∗∗^*p* < 0.01, n.s.- ANOVA did not stand Bonferroni correction (*p* > 0.05 for the main effect).

We next determined whether DHEA supplement intervention would alter the state of methylation of gene promoters in neurobiological-related pathways in the addicted group. We focused on the state of methylation of these particular sites in our Illumina 450K arrays. One-way ANOVA with Bonferroni correction revealed an effect of treatment (adjusted *p* < 0.05) on genes that were hypermethylated in addicts and became hypomethylated after DHEA treatment (ITGB2, LCK, ITGA6, MAPK13, and FASLG; **Figures 3A,B**; see **Supplementary Table 4** for statistics, degrees of freedom, and effect sizes). Genes that were hypomethylated in the addicted group (ALDH4A1, DHRS9, ALDH3B1, ALDH3A1, IL4I1, DHRS4, and CSGALNACT1) were re-methylated in the DHEA treated group, though this difference did not reach significance with the exception of the gene DHRS9 (**Figure 3C**). These results are consistent with the idea that the differential methylation of neurobiological-related genes in addicts is reversed when addiction is reversed.

### DNA Methylation Levels Correlated With Negative Affect Score and Decision Making

In our previous work, DHEA-treated patients showed a significant improvement (*p* = 0.04 for interaction DHEA X time) in the negative affect score of the Positive and Negative Affect Schedule (PANAS) test, 1 month after treatment initiation (Ohana et al., 2016).

**FIGURE 4:**
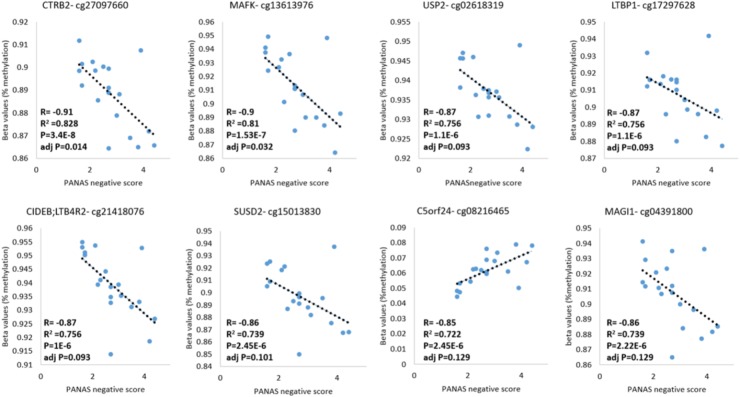
DNA methylation levels of CG sites that correlate with the PANAS negative affect score. Probes significantly correlated with PANAS negative affect score of addicts are depicted (*p* < 0.05; FDR < 0.2).

DNA methylation and behavioral phenotypes are quantitative traits. Therefore, we analyzed the correlation between DNA methylation levels and the negative score of the PANAS tests across samples acquired from addicts who filled-in the PANAS questionnaire, to identify methylation sites that are possibly involved in these phenotypes. A Pearson’s correlation analysis found 14 probes that are linearly correlated to the PANAS negative affect score of addicts after correction for multiple testing (*p* < 0.05; FDR < 0.2, **Figure 4** and **Supplementary Table 5**). Out of these probes, the top eight were annotated to known genes: *CTRB2, MAFK, USP2, LTBP1, LTB4R2/CIDEB, SUSD2, C5ORF24*, and *MAGI*. In an additional individual analyses, two of these probes were significantly differently methylated between healthy and placebo-treated patients at baseline with one hypo-methylated and one hyper-methylated (**Supplementary Figure 1**). However, some of these sites were not different between addicts and controls suggesting that they are unrelated to addiction but related to negative affect as measured by the PANAS test. Since we don’t have data for controls on this behavior our ability to interpret these data is limited.

The Iowa Gambling Task score, a measure of decision making, was improved following DHEA treatment in patients from our previous study (*p* = 0.01 for within DHEA group comparison). A correlation analysis between Iowa gambling score and DNA methylation levels resulted in 2,731 probes which were significantly correlated (**Supplementary Table 6**). However, no probes passed FDR correction (best un-corrected *p*-value = 9.92E-05; *r* = -0.774). This suggests that DNA methylation may contribute to decision-making performance. However, since no probe stands FDR-correction it is possible that our present study is under-powered to detect correlation between DNA methylation and decision-making. A larger cohort may be required to attain sufficient power to confirm this association.

### Overlap Between Hypo-methylated Genes in Addicts T-Cell and Rat Nucleus Accumbens

**FIGURE 5:**
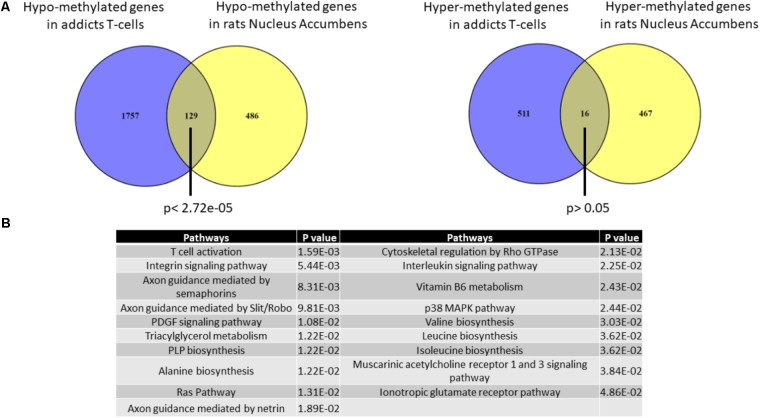
Overlap between genes that are differentially methylated in T-cells from addicts and differentially methylated genes in cocaine-intake trained-rats nucleus accumbens. **(A)** Lists of differentially methylated genes in addicts T-cells and cocaine self-administration trained-rats nucleus accumbens were compared. A significant overlap was found for hypo-methylated (left) but not hyper-methylated (right) genes using Fisher hypergeometric test. **(B)** Table of pathways significantly over-represented in the list of genes commonly hypo-methylated in human T-cells and rat nucleus accumbens.

It is impossible to study in living humans DNA methylation in brain regions that are involved in the addictive phenotype such as the Nucleus Accumbens (Nac). We therefore used an across-species approach to examine the correspondence between DNA methylation differences that are associated with addiction in T cells and the brain. We analyzed the degree of overlap between the gene list of differentially methylated regions in addicts and the gene list of differentially methylated regions in the NAc of rats trained to self-administer cocaine (Accession: GSE66350: GSMs: 1620076-8 and GSMs: 1620080-2). A significant overlap was found between the two tissues for hypo-methylated but not hyper-methylated genes (129 genes- *p* < 2.72E-5 and 16 genes- *p* > 0.05, respectively, hypergeometric test; **Figure 5A**). The overlapping hypo-methylated genes were enriched for pathways related mainly to T-cell activation and interestingly also to axonal guidance (**Figure 5B**).

### Overlap Between Differentially Methylated CpG in Alcohol Users and Current Findings

**FIGURE 6:**
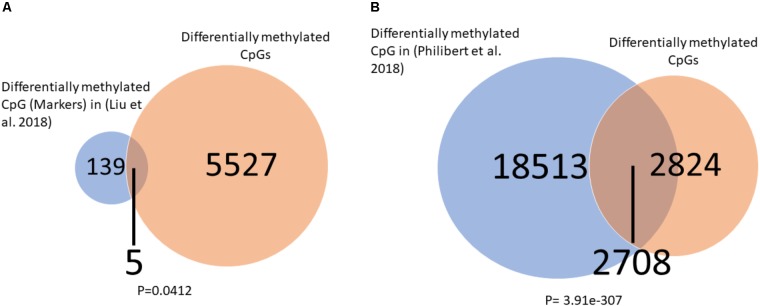
Overlap between differentially methylated CpG in alcohol users and current findings. **(A)** A significant overlap was observed between our list of differentially methylated CpGs and a list of alcohol use markers found by Liu et al. (2018). **(B)** A significant overlap was observed between our list of differentially methylated CpGs and a list of alcohol use markers found by Philibert et al. (2018). Significance was assessed by the Fisher hyper-geometric test.

To further explore the relation of our findings to recent finding in the literature we overlapped our significantly differentially methylated CpGs (healthy control vs. placebo at baeline) to those of two other studies which measured DNA methylation in alcohol users using the same Illumina 450K arrays. We found (**Figures 6A,B**) a significant overlap between our findings and those of others (Liu et al., 2018; Philibert et al., 2018) suggesting a common DNA methylation landscape across addiction to different substances.

## Discussion

Previous studies have shown a broad signature of addiction in DNA methylation in the NAc of rats during cocaine craving (Massart et al., 2015), however, it is unknown whether addiction is associated with a similar altered DNA methylation signature in humans. DNA methylation biomarkers could serve as early predictors of addiction susceptibility, as well as measures of response to interventions. Moreover, if DNA methylation profiles are indeed altered in addicts, this could provide a rationale for considering epigenetic based interventions. However, since it is impossible to measure DNA methylation in the brain in living humans, it is critical to determine whether informative DNA methylation profiles exist in non-invasive sources such as peripheral white blood cells. In this study, we focused on T cells to reduce the confounding effects of a mixed cell population that is present in white blood cells. In addition, T cells play an important role in immune system memory and coordination of cytokine secretion which are known to affect CNS functioning (Deverman and Patterson, 2009; Lisak et al., 2011).

The results of our analysis using a limited number of subjects is consistent with the hypothesis that addiction is associated with a DNA methylation signature in peripheral immune cells such as T cells, and is correlated with measures of negative affect. Further, in a separate analysis we pooled data across groups to explore the possible link between DNA methylation and mood (measured by the PANAS negative test) in the population of drug addicts. Notably, several probes that are correlated to the PANAS negative affect score are of special interest: *MAFK* and *SUSD2* are involved in regulation of neurite growth (Kataoka et al., 1995; Nadjar et al., 2015), peripheral blood *USP2* expression is correlated to positive psychosis symptoms (Bousman et al., 2010), copy number variants of *LTBP1* are associated with alcohol drinking (Pei et al., 2012) and a single-nucleotide polymorphism in the scaffold protein *MAGI1* is associated with major depression and neuroticism (Yamagata and Sanes, 2010; Genetics of Personality Consortium et al., 2015). Taken together, these findings suggest that quantitative differences in DNA methylation of these genes in drug addicts correlate with quantitative differences in negative affect, pointing to a putative role for DNA methylation in these phenotypes.

Interestingly, the differentially methylated sites are overwhelmingly hypomethylated, consistent with a coordinated mechanism of change in DNA methylation. Other pathological conditions such as cancer (Stefanska et al., 2011) and Lupus autoimmunity (Yung and Richardson, 1994; Absher et al., 2013) involve hypomethylation of a broad swath of genes. Hypomethylation of promoters is usually associated with activation or poising of promoter for activation, and therefore the data suggest activation of these pathways in addicted individuals.

Analysis of the pathways that are differentially methylated in addiction reveal two interesting categories, i.e., immune and inflammatory functions as well as neurobiological-related pathways such as reelin and corticotrophin signaling which were hyper methylated and several neurotransmitter degradation pathways which were hypomethylated. The immune involvement might reflect the poor physical state of addicts or might be a component of the addicted phenotype itself. Inflammatory functions were associated with other neuropsychiatric disorders such as Alzheimer’s disease (Agostinho et al., 2010).

Similarly, the neurobiological pathways involve genes that are related to neurotransmitter regulation which might represent an adaptation to the addicted state. Alternatively, these genes might play a causal role in the addictive phenotype. These questions are difficult to resolve in human studies and could be addressed using DNA methylation targeted interventions or in animal models. Causality is difficult to prove in humans but could be tested in animal models by examining whether expression of differentially methylated genes has an impact on the addictive phenotype by either knockdown or over expression of these genes. In addition, the role of DNA methylation could be evaluated by targeted epigenetic editing or by using DNA methylation inhibitors or stimulators. We recently demonstrated that DNA methylation inhibitor or activator treatments alter cocaine craving phenotypes in rats (Massart et al., 2015). However, these studies did not examine peripheral DNA methylation states and these remain to be examined.

To determine whether DNA methylation profiles of neurobiological-related genes are tightly associated with the addicted phenotype we measured the state of methylation of these sites after an intervention with DHEA, a supplement that we have previously shown to have a long-term therapeutic effect on human addicts (Ohana et al., 2016). The results suggest that DHEA treatment in addicts reverses the difference in DNA methylation in genes relevant to brain function and behavior. Neuronal related genes that were hypermethylated in addicted persons became less methylated in the DHEA treated group, while genes that were hypomethylated in addicts became more methylated with DHEA treatment. Although these data are consistent with the hypothesis that the methylation state of the genes is tightly associated with the state of addiction, these data do not rule out the possibility that DNA methylation of these sites is a consequence rather than cause of addiction.

One of the most persistent and challenging question in behavioral epigenetics is the relevance of peripheral DNA methylation markers to phenotypes that are mainly regulated by particular brain regions. It is very difficult to address such a question in humans since the brain is inaccessible to methylation analysis in living humans. We therefore used an evolutionary approach and determined whether there was an overlap between differentially methylated genes in T cells in humans and genes that we have recently found to be differentially methylated in NAc, a brain region involved in addiction in a rat model of cocaine craving (Massart et al., 2015). The significant overlap of hypomethylated gene between the two tissues across species supports the relevance of these differentially methylated sites for the addictive phenotype. These results add to the increasing body of data that support the idea that peripheral T cells are highly informative on epigenetic changes that occur in the brain and are associated with behavioral phenotypes (Massart et al., 2016). It should be, however, noted that the strong inflammatory signal detected in the differentially methylated profile suggests that there are system wide components to behavioral phenotypes in the immune system.

Important limitations of our study are the small number of samples and the comparison between poly-drug users to rats treated with only cocaine under a fixed-dose intake. However, the effect sizes are very large and the pooled sigma is relatively small (average of 0.049). For example, power calculations show that a sample of only nine samples was sufficient to detect a delta beta of 0.1 with power of 0.8 taking into account a pooled sigma of 0.049 and a FDR threshold of 0.05 (unadjusted *p* < 1 × 10^-3^; based on the real numbers of adjusted FDR and unadjusted *p*-values in this study). For effect sizes larger than 2 the sample size needed is even smaller. We recognize that non-random bias and stratification is common with small sample sizes and therefore it is important to replicate this study. However, power calculations indicate that increasing the sample size beyond 10 will not increase power for detection of the top differentially methylated CGs. For example, detection of delta beta of 0.15 with pooled sigma of 0.045 (average of our top 20 CGs) at a Bonferroni corrected *p*-value of 1E-7 will reach a power close to 1 with a sample size of 10 and power will not increase significantly beyond this number.

Although the number of subjects in our study is small, the combination of the results presented here, the correlation between quantitative distribution of methylation of several sites with quantitative differences in behavior, and the reversal of methylation states of several genes with an intervention that reduces addictive behaviors, points to a functional role for these differences in DNA methylation in addiction.

We further confirmed our study by intersecting our markers for addiction with those found by two other studies which focused on alcohol use (Liu et al., 2018; Philibert et al., 2018). The fact that significant overlaps were found further confirm a common epigenetic signature of drug abuse in peripheral blood samples.

Our study provides preliminary evidence and justification for further studies with a larger number of subjects with wider inclusion criteria to establish associations of addiction with DNA methylation, as well as the effects of different interventions on the state of methylation. These studies should further confirm association of DNA methylation in peripheral white blood cells, and facilitate delineation of sites that predict both drug craving and response to treatment. Establishing a causal link between DNA methylation and addiction will point to epigenetic therapeutics in reprogramming the addicted state.

## Availability of Data and Materials

GEO accession number for the raw IDAT files is: https://www.ncbi.nlm.nih.gov/geo/query/acc.cgi?acc=GSE118570.

## Author Contributions

EL performed the data analysis and interpretation of findings. GW isolated T-cells DNA from patients’ samples. YD, PR, AP, and AW contributed to the study concept and design. DO collected the samples and the cognitive tests data with guidance from RM. GY and MS were responsible for the study concept and design. EL and MS wrote the manuscript. All authors critically reviewed content and approved final version for publication.

## Conflict of Interest Statement

The authors declare that the research was conducted in the absence of any commercial or financial relationships that could be construed as a potential conflict of interest.

## Footnotes

1 http://bioinfogp.cnb.csic.es/tools/venny/index.html
