# Supplementary material for: A DNA Methylation Signature of Addiction in T Cells and Its Reversal With DHEA Intervention
Source: Front Mol Neurosci. 2018 Sep 10;11:322. doi: 10.3389/fnmol.2018.00322 (PMC6139343; doi:10.3389/fnmol.2018.00322)
Supplement: Supplementary file 2 [file Table_2.docx]

**Payoff structure of the Iowa Gambling task**:

| Deck | Wins | Losses | Deck category |
| --- | --- | --- | --- |
| A | 100 every trial | In each trial, 50% chance of losing 200, 250, or 300 (with equal chance) | Disadvantageous |
| B | 100 every trial | In each trial, 10% chance of losing 1250 | Disadvantageous |
| C | 50 every trial | In each trial, 50% chance of losing 25,50 or 75 (with equal chance) | Advantageous |
| D | 50 every trial | In each trial, 10% chance of losing 250 | Advantageous |
