# Supplementary figures and images for: A DNA Methylation Signature of Addiction in T Cells and Its Reversal With DHEA Intervention
Source: Front Mol Neurosci. 2018 Sep 10;11:322. doi: 10.3389/fnmol.2018.00322 (PMC6139343; doi:10.3389/fnmol.2018.00322)

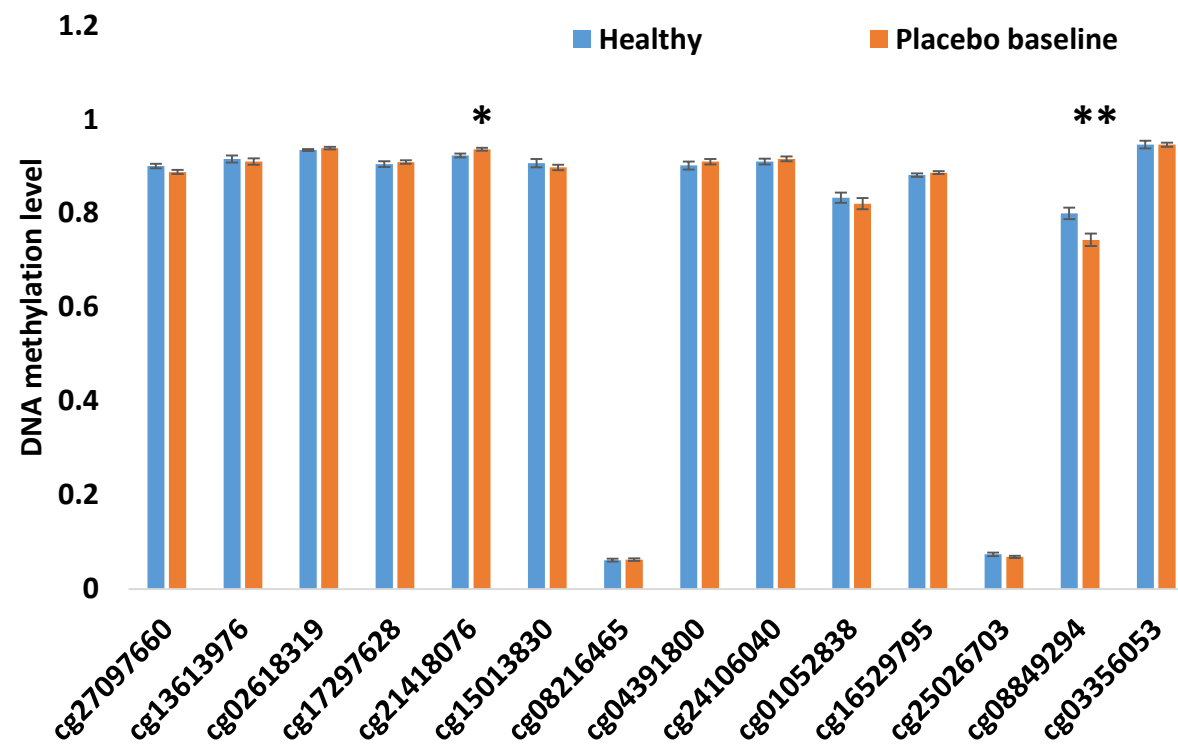

Supplement: Supplementary file 7 [file Data_Sheet_1.PDF]
