# Supplementary material for: A DNA Methylation Signature of Addiction in T Cells and Its Reversal With DHEA Intervention
Source: Front Mol Neurosci. 2018 Sep 10;11:322. doi: 10.3389/fnmol.2018.00322 (PMC6139343; doi:10.3389/fnmol.2018.00322)
Supplement: Supplementary file 8 [file Data_Sheet_2.pdf]

Supplementary Material A DNA Methylation Signature of Addiction in T cells and Its Reversal with DHEA Intervention

Elad Lax, Gal Warhaftig, David Ohana, Rachel Maayan, Yael Delayahu, Paola Roska, Alexander M. Ponizovsky, Abraham Weizman, Gal Yadid*, Moshe Szyf

* Correspondence: Corresponding Author: yadidg@gmail.com

Supplementary Figure 1: A graph depicting DNA methylation levels in healthy and placebotreated patients at baseline for 14 CpGs which were significantly correlated with PANAS negative score in addicts. (T-tests; *p<0.05, **p<0.01, not-FDR-corrected).

Supplementary Table 1: Individual drug intake information.

Supplementary Table 2: Payoff structure of the Iowa Gambling task.

Supplementary Table 3: List of CpGs differentially methylated in Nicotine smokers.

Supplementary Table 4: Statistics for DHEA effect on DNA methylation.

Supplementary Table 5: Illumina probe ID correlation statistics and gene annotation for probes significantly correlated with negative PANAS score.

Supplementary Table 6: Correlation statistics between DNA methylation and Iowa Gambling Score
